# Large Language Model–Assisted Surgical Consent Forms in Non-English Language: Content Analysis and Readability Evaluation

**DOI:** 10.2196/73222

**Published:** 2025-06-19

**Authors:** Namkee Oh, Jongman Kim, Sunghae Park, Sunghyo An, Eunjin Lee, Hayeon Do, Jiyoung Baik, Suk Min Gwon, Jinsoo Rhu, Gyu-Seong Choi, Seonmin Park, Jai Young Cho, Hae Won Lee, Boram Lee, Eun Sung Jeong, Jeong-Moo Lee, YoungRok Choi, Jieun Kwon, Kyeong Deok Kim, Seok-Hwan Kim, Gwang-Sik Chun

**Affiliations:** 1 Department of Surgery, Samsung Medical Center, 81 Ilwonro, Seoul, Republic of Korea, 82 1093650277; 2 Department of Surgery, Seoul National University Bundang Hospital, Seongnam-si, Republic of Korea; 3 Department of Surgery, Dongguk University Ilsan Hospital, Goyang-si, Republic of Korea; 4 Department of Surgery, Seoul National University Hospital, Seoul, Republic of Korea; 5 Department of Surgery, Soon Chun Hyang University Cheonan Hospital, Cheonan, Republic of Korea; 6 Department of Surgery, Inha University Hospital, Incheon, Republic of Korea; 7 Department of Surgery, Chungnam National University, Daejeon, Republic of Korea

**Keywords:** informed consent, natural language processing, surgical procedures, operative, large language model, readability, ChatGPT-4o, surgical consent form, liver resection

## Abstract

**Background:**

Surgical consent forms convey critical information; yet, their complex language can limit patient comprehension. Large language models (LLMs) can simplify complex information and improve readability, but evidence of the impact of LLM-generated modifications on content preservation in non-English consent forms is lacking.

**Objective:**

This study evaluates the impact of LLM-assisted editing on the readability and content quality of surgical consent forms in Korean—particularly consent documents for standardized liver resection—across multiple institutions.

**Methods:**

Standardized liver resection consent forms were collected from 7 South Korean medical institutions, and these forms were simplified using ChatGPT-4o. Thereafter, readability was assessed using KReaD and Natmal indices, while text structure was evaluated based on character count, word count, sentence count, words per sentence, and difficult word ratio. Content quality was analyzed across 4 domains—risk, benefit, alternative, and overall impression—using evaluations from 7 liver resection specialists. Statistical comparisons were conducted using paired 2-sided *t* tests, and a linear mixed-effects model was applied to account for institutional and evaluator variability.

**Results:**

Artificial intelligence–assisted editing significantly improved readability, reducing the KReaD score from 1777 (SD 28.47) to 1335.6 (SD 59.95) (*P*<.001) and the Natmal score from 1452.3 (SD 88.67) to 1245.3 (SD 96.96) (*P*=.007). Sentence length and difficult word ratio decreased significantly, contributing to increased accessibility (*P*<.05). However, content quality analysis showed a decline in the risk description scores (before: 2.29, SD 0.47 vs after: 1.92, SD 0.32; *P*=.06) and overall impression scores (before: 2.21, SD 0.49 vs after: 1.71, SD 0.64; *P*=.13). The linear mixed-effects model confirmed significant reductions in risk descriptions (β₁=−0.371; *P*=.01) and overall impression (β₁=−0.500; *P*=.03), suggesting potential omissions in critical safety information. Despite this, qualitative analysis indicated that evaluators did not find explicit omissions but perceived the text as overly simplified and less professional.

**Conclusions:**

Although LLM-assisted surgical consent forms significantly enhance readability, they may compromise certain aspects of content completeness, particularly in risk disclosure. These findings highlight the need for a balanced approach that maintains accessibility while ensuring medical and legal accuracy. Future research should include patient-centered evaluations to assess comprehension and informed decision-making as well as broader multilingual validation to determine LLM applicability across diverse health care settings.

## Introduction

Informed consent is a primary legal and ethical requirement for surgical and other invasive procedures, ensuring that patients fully understand the benefits, risks, and alternatives before making medical decisions [1,2]. Despite its importance, the informed consent process remains challenging in clinical practice due to the complexity of the medical information, difficulty of medical terminology, and asymmetry of knowledge between health care professionals and patients [2,3]. These challenges often lead to poor patient comprehension and increased medical disputes related to the duty of explanation [4,5].

Recent studies have explored the potential of generative artificial intelligence (AI), particularly large language models (LLMs), to improve the readability of informed consent documents [6-8]. LLMs demonstrate a promising ability to simplify complex medical texts, potentially making them more accessible to patients. However, although preliminary studies confirm gains in readability, crucial questions remain regarding whether the essential qualitative integrity and nuanced meaning of the original consent documents are adequately preserved during AI-driven simplification. Furthermore, existing research has predominantly focused on English-language documents, leaving the applicability and content fidelity of LLMs in different linguistic and cultural contexts largely unexplored, particularly concerning the preservation of medicolegal nuance specific to the diverse health care system [9]. To our knowledge, this study is among the first to quantitatively assess the impact of LLM-assisted editing applied to non-English (Korean) surgical consent forms on both readability and content preservation, aiming to provide a valuable benchmark for future research.

Therefore, this study aims to address these gaps by evaluating both the readability enhancement and the preservation of content integrity in LLM-edited surgical consent forms for liver resection by using standardized documents from multiple Korean institutions. By systematically assessing these 2 key dimensions, this research seeks to contribute to a deeper understanding of the potential benefits and inherent limitations of deploying LLMs for consent documentation in multilingual clinical settings, informing their responsible development and potential application in real-world medical practice.

## Methods

### Data Collection

Standardized liver resection consent forms were collected from 7 medical institutions in South Korea (Chungnam National University Hospital, Inha University Hospital, Dongguk University Ilsan Hospital, Samsung Medical Center, Seoul National University Hospital, Seoul National University Bundang Hospital, and Soonchunhyang University Seoul Hospital). These institutions were selected to represent a diverse range of hospital settings to ensure the generalizability of the findings. The consent forms were official documents used in clinical practice, and no modifications were made prior to AI-based editing.

### LLM-Based Text Simplification

Each collected consent form was simplified using ChatGPT-4o with the following prompt: “The following is a surgical consent form used in hospitals to explain liver resection to patients. Please rewrite it in a way that a first-year middle school student (7th grade) can easily comprehend while ensuring that all essential information is retained.” The ChatGPT-4o model was used without domain-specific fine-tuning. The AI-generated consent forms were directly compared with the original version for readability and content quality.

### Readability and Text Structure Analysis

To assess the impact of LLM-based simplification, we conducted readability and structural comparisons between the original (before) and AI-edited consent (after) forms. Readability was quantitatively measured using KRead and Natmal—the 2 established readability indices for the Korean language [10,11]. These indices were chosen due to their ability to quantify linguistic complexity at different comprehension levels. In addition to readability, we performed a quantitative text analysis, comparing the before and after versions across the following metrics: character count, word count, sentence count, words per sentence, and difficult word ratio (percentage of words classified as difficult by Korean linguistic databases). These measures allow for an objective comparison of linguistic complexity beyond readability scores.

### Content Quality Assessment

To evaluate whether LLM-assisted modification preserved the essential content, we assessed the consent forms by using 4 quality domains adapted from Decker et al [7]: (1) risk—completeness and accuracy of the risk explanations; (2) benefit—clarity and comprehensiveness of the expected benefits; (3) alternative—presentation of alternative treatment options; and (4) overall impression—general coherence and effectiveness of the consent document. Each consent form was evaluated independently by 7 liver resection specialists, with at least 2 evaluators per document. Evaluators were blinded to the original document (AI-edited vs original) to minimize bias in subjective scoring.

### Statistical Analysis

Statistical analyses were conducted to evaluate the impact of LLM-generated modifications on readability and content quality. Descriptive statistics (mean and standard deviation) were computed for readability metrics (KReaD, Natmal) and text structure features (characters, words, sentences, words per sentence, difficult word ratio). A paired 2-sided *t* test was conducted to compare the quality scores (risk, benefit, alternative, overall) between the original and LLM-edited versions to determine whether significant differences existed. To account for the variability in institutional policies and evaluator subjectivity, we applied the linear mixed-effects model. In this model, the document version (original vs AI-edited) was treated as a fixed effect, while the institution and the evaluator were treated as random effects to control for scoring heterogeneity. The linear mixed-effects model assumes linearity, independence of errors, homoscedasticity, and normality of residuals. While accounting clustering, sensitivity to these assumptions represents a potential limitation of the model. All statistical tests were 2-tailed, with a significance threshold of *P*<.05. Analyses were conducted using Python (SciPy, statsmodels, Seaborn, Matplotlib).

## Results

### Impact of LLM-Based Simplification

Table 1 provides a summary of the impact of LLM-based simplification on text structure, readability, and content quality assessment. Content quality was analyzed across 4 domains: risk, benefit, alternative, and overall impression.

**Table 1.** Effect of large language model–assisted editing on text structure, readability, and content quality of surgical consent forms.

	Before LLM^a^-assisted editing, mean (SD)	After LLM-assisted editing, mean (SD)	*P* value
Text structure			
Character count	6074.14 (170.33)	4241.00 (594.73)	.03
Word count	1486.57 (534.99)	1113.43 (163.67)	.047
Sentence count	106.86 (35.36)	120.57 (33.74)	.11
Words per sentence	15.01 (5.13)	9.23 (4.85)	<.001
Difficult word ratio	0.77 (0.06)	0.49 (0.04)	<.001
Readability (lower=easier)			
KReaD score	1777 (28.47)	1335.6 (59.95)	<.001
Natmal score	1452.3 (88.67)	1245.3 (96.96)	.007
Risk, benefit, alternative, overall impression quality assessment			
Risk	2.29 (0.47)	1.92 (0.32)	.06
Benefit	2.14 (0.56)	2.07 (0.45)	.80
Alternative	2.14 (0.61)	1.93 (0.67)	.51
Overall impression	2.21 (0.49)	1.71 (0.64)	.13

a LLM: large language model.

### Text Structure Modifications

**Figure 1.**
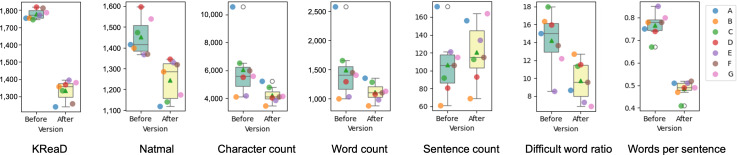
Impact of large language model–assisted editing on readability and text structure metrics of Korean surgical consent forms across institutions. This figure presents comparisons of the readability and text structure metrics before and after large language model–assisted modifications across multiple institutions (A-G). The box plots display the distribution of values for each metric. KReaD and Natmal scores represent readability, with lower values indicating improved readability. The character count and word count reflect text length reductions after artificial intelligence simplification. The sentence count, difficult word ratio, and words per sentence illustrate the structural modifications affecting linguistic complexity. Each colored dot corresponds to a specific institution (A-G), providing an institution-wise breakdown of artificial intelligence–driven changes.

LLM editing resulted in significant changes to the text structure. There was a significant reduction in character count (mean decrease of 1833 characters, from 6074.14, SD 2170.33 to 4241.00, SD 594.73; *P*=.03) and word count (mean decrease of 373 words, from 1486.57, SD 534.99 to 1113.43, SD 163.67; *P*=.047). Although the total number of sentences did not change significantly (106.86, SD 35.36 vs 120.57, SD 33.74; *P*=.11), sentences became considerably shorter, with words per sentence decreasing significantly from 15.01 (SD 5.13) to 9.23 (SD 4.85) (*P*<.001). The proportion of difficult words also showed a significant reduction (0.77, SD 0.06 vs 0.49, SD 0.04; *P*<.001), indicating simpler vocabulary usage. Institution-specific text structure changes are detailed in Table 2 and Figure 1.

**Table 2.** Institution-specific (A-G) analysis of large language model–assisted modifications in text structure and readability of surgical consent forms.

		A	B	C	D	E	F	G	Mean (SD)	*P* value
Text structure										
Character count										.03
Before		10,566	4121	6539	5519	4202	5980	5592	6074.14 (2170.33)	
After		5241	3459	4798	4066	3859	4123	4141	4241.00 (594.73)	
Word count										.047
Before		2581	996	1652	1291	1034	1449	1403	1486.57 (534.99)	
After		1349	875	1284	1073	982	1102	1129	1113.43 (163.67)	
Sentence count										.11
Before		172	61	92	81	121	106	115	106.86 (35.36)	
After		156	69	113	93	134	115	164	120.57 (33.74)	
Words per sentence										<.001
Before		15.01	16.33	17.96	15.94	8.55	13.67	12.20	13.91 (15.13)	
After		8.65	12.68	11.36	11.54	7.33	9.58	6.88	9.23 (4.85)	
Difficult word ratio										<.001
Before		0.75	0.78	0.67	0.74	0.85	0.78	0.8	0.77 (0.06)	
After		0.51	0.47	0.41	0.49	0.51	0.52	0.49	0.49 (0.04)	
Readability (lower=easier)										
KReaD										<.001
Before		1754	1754	1746	1817	1772	1811	1785	1777.00 (28.47)	
After		1244	1360	1336	1370	1397	1261	1381	1335.57 (59.95)	
Natmal										.007
Before		1417	1400	1475	1597	1367	1370	1540	1452.29 (88.67)	
After		1120	1285	1140	1347	1330	1320	1175	1245.29 (96.96)	

### Readability Improvements

Consistent with the structural modifications, the readability of the consent forms significantly improved following LLM editing. The mean KReaD readability score decreased from 1777 (SD 28.47) to 1335.6 (SD 59.95) (*P*<.001), and the mean Natmal score decreased from 1452.3 (SD 88.67) to 1245.3 (SD 96.96) (*P*=.007). Both indices indicate a significant reduction in linguistic complexity, suggesting improved accessibility for patients with reading levels potentially equivalent to a middle-school reading level. Table 2 and Figure 1 provide institution-wise readability results.

### Content Quality Assessment: Expert Evaluations

**Figure 2.**
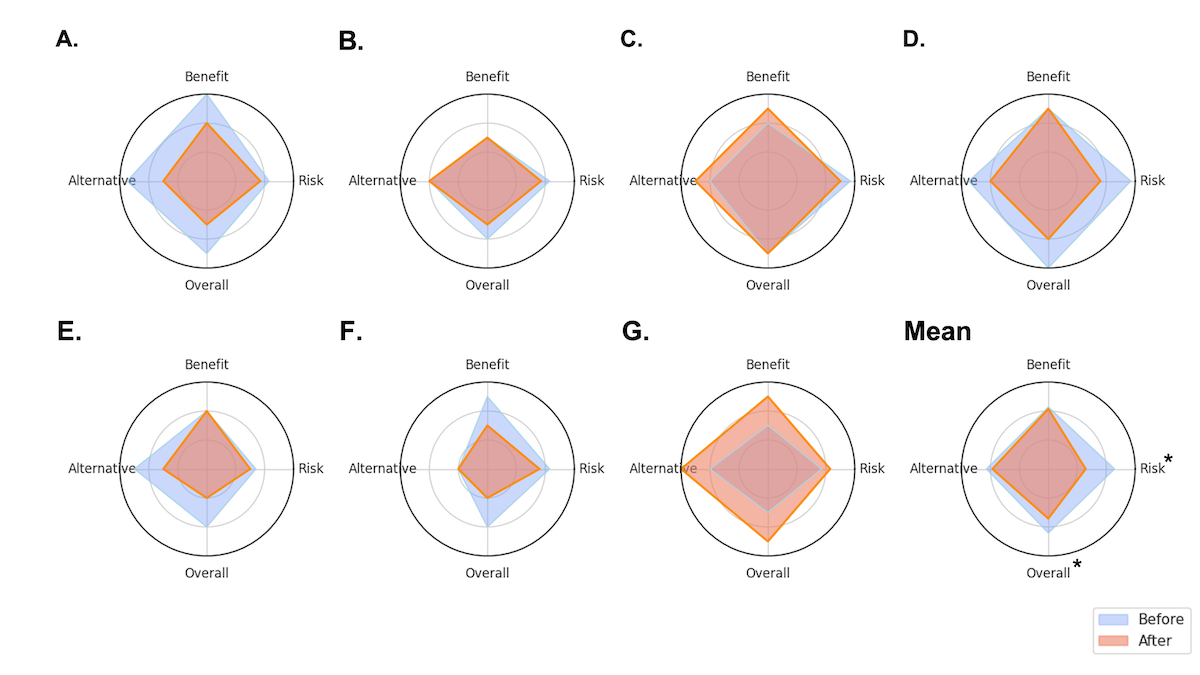
Comparison of risk, benefit, alternative, and overall scores before and after large language model–assisted editing across institutions*.* This figure presents the radar plots comparing risk, benefit, alternative, and overall impression scores before (blue) and after (orange) large language model–assisted editing for surgical consent forms. Panels A-G represent individual institutions. The mean panel shows the aggregated results across all institutions. Asterisks (*) indicate statistically significant decreases in risk and overall scores (*P*<.05) after artificial intelligence modification. The blue shaded area represents original consent forms, while the orange shaded area represents artificial intelligence–edited consent forms, highlighting observed changes in content quality.

Expert evaluation using the risk, benefit, alternative, overall impression scoring system revealed trends toward lower scores after LLM editing, particularly for risk descriptions (mean score change from 2.29, SD 0.47 to 1.92, SD 0.32) and overall impression (2.21, SD 0.49 to 1.71, SD 0.64). However, the paired 2-sided *t* tests comparing the mean scores before and after editing across all institutions showed that these decreases did not reach statistical significance at *P*<.05 (risk: *P*=.06; overall: *P*=.13). Similarly, scores for benefit explanations (2.14, SD 0.56 vs 2.07, SD 0.45; *P*=.81) and alternative treatment descriptions (2.14, SD 0.61 vs 1.93, SD 0.67; *P*=.52) showed no significant changes in this analysis. Institution-specific risk, benefit, alternative, and overall impression scores are presented in Table 3 and Figure 2.

**Table 3.** Institution-specific (A-G) changes in risk, benefit, alternative, and overall impression scores after large language model–assisted consent form editing.

		A	B	C	D	E	F	G	Overall	*P* value
Risk, mean (SD)										.06
Before		2.5 (0.28)	2.15 (0.21)	2.85 (0.21)	2.85 (0.21)	1.7 (0)	2.15 (0.21)	1.85 (0.21)	2.29 (0.47)	
After		1.85 (0.21)	1.85 (0.21)	2.5 (0.71)	1.80 (0.71)	1.5 (0.28)	1.8 (0.71)	2.15 (0.21)	1.92 (0.32)	
Benefit, mean (SD)										.81
Before		3 (0)	1.5 (0.71)	2 (0)	2.5 (0.71)	2 (0)	2.5 (0.71)	1.5 (0.71)	2.14 (0.56)	
After		2 (0)	1.5 (0.71)	2.5 (0.71)	2.5 (0.71)	2 (0)	1.5 (0.71)	2.5 (0.71)	2.07 (0.45)	
Alternative, mean (SD)										.52
Before		2.75 (0.35)	2 (0)	2 (0.71)	2.75 (0.35)	2.5 (0.71)	1 (0)	2 (0.71)	2.14 (0.61)	
After		1.5 (0.71)	2 (0.71)	2.5 (0)	2 (1.41)	1.5 (0)	1 (0)	3 (0)	1.93 (0.67)	
Overall, mean (SD)										.13
Before		2.5 (0.71)	2 (0)	2.5 (0.71)	3 (0)	2 (0)	2 (0)	1.5 (0.71)	2.21 (0.49)	
After		1.5 (0.71)	1.5 (0.71)	2.5 (0.71)	2 (0)	1 (0)	1 (0)	2.5 (0.71)	1.71 (0.64)	

### Linear Mixed-Effects Model Analysis of Content Quality

To account for the potential variability between institutions and individual evaluator scoring patterns, we conducted a linear mixed-effects model analysis. This sensitive analysis revealed a statistically significant decrease in the expert ratings for risk descriptions after LLM editing (β₁=−0.371; *P*=.01) and for overall impression (β₁=−0.500; *P*=.03), even after controlling for the random effects. In contrast, no significant differences were found using the linear mixed-effects model for benefit explanations (β₁=−0.071; *P*=.76) or alternative treatment options (β₁=−0.214; *P*=.39).

## Discussion

### Principal Findings

This study evaluates the impact of LLM-assisted editing on the readability and content quality of Korean surgical consent forms across multiple institutions. Our findings demonstrate that LLM simplification significantly improved the readability scores (KReaD, Natmal) and reduced the text complexity (shorter sentences, fewer difficult words), potentially making consent forms more accessible, aligning with a middle-school reading level. However, this gain in simplicity was accompanied by a statistically significant decrease in expert-rated quality for risk descriptions and overall impression, as confirmed by the linear mixed-effects model analysis controlling for institutional and evaluator variability. The benefits and alternative explanations remained largely unchanged. These findings suggest that although AI-enhanced consent forms offer significant accessibility gains, careful consideration of content integrity, perceived professionalism, and medicolegal validity is crucial.

The findings of this study align with previous research demonstrating that LLMs can enhance the readability of medical texts but also raise questions about content integrity [6,7,9]. Ali et al [6] reported that GPT-4 significantly simplified surgical consent forms, improving readability levels from college-level text to an eighth grade level. However, their study did not quantify the potential loss of information, leaving concerns about content accuracy unaddressed. Decker et al [7] further showed that LLM-generated informed consent documents were more readable and complete than those written by surgeons, but they did not account for the variability among physicians. Our study builds upon these findings by incorporating structured quality assessments (risk, benefit, alternative, and overall impression scoring) and linear mixed-effects modeling to evaluate not only readability but also content retention across institutions. Unlike previous studies, this research controlled for institutional and evaluator variability, making the results more generalizable to real-world clinical settings.

The significant decline in risk descriptions and overall impression scores, despite improvements in objective readability metrics, warrants careful interpretation [8,12]. A closer qualitative examination of the text modification, particularly in the sections describing risks for the consent form exhibiting the most substantial decrease in the risk score, reveals how simplification affected perceived content integrity. Although an LLM generally preserved the core list of potential complications, it often omitted specific details regarding their context, consequences, management, and severity, which likely contributed to lower expert ratings. For example, explanations for major risks such as liver failure or ascites often lost specifics regarding management nuances, indicators of potential severity (such as high probability in certain conditions or downstream effects like electrolyte imbalance), and preventative context. From an expert perspective, this reduction in detail and nuance likely rendered the descriptions less complete, potentially understating the risks or appearing less professional, thus negatively impacting the perceived content integrity crucial for surgical consent [13]. This highlights the core challenge: achieving patient-friendly readability without sacrificing the clinical precision and comprehensive information deemed essential by health care professionals.

This underscores the critical need to move beyond readability scores and expert ratings toward patient-centered evaluations. Future research must directly assess patient comprehension by using validated methods such as postconsent comprehension questionnaires. Comparing actual patient understanding derived from the original version versus patient understanding of LLM-simplified forms is paramount to determine if enhanced readability translates into truly informed decision-making and meets diverse health literacy needs. Establishing the clinical validity of these tools requires measuring their impact on the end user (the patients).

Furthermore, deploying LLM-simplified or LLM-generated consent forms necessitates careful legal and ethical considerations. In the Korean legal context, informed consent requires fulfilling the duty of explanation, demanding sufficient detail on the risks, benefits, and alternatives. Oversimplification, even if unintentional, could render a consent form legally inadequate if critical nuances are lost or the document’s tone undermines the perceived seriousness required by law. Additionally, using external LLM services raises data privacy and security concerns that must comply with regulations such as the Personal Information Protection Act. Ethically, the goal must be genuine patient understanding—not merely simplified text; thus, rigorous validation against both specific legal standards and patient comprehension benchmark is an ethical prerequisite before clinical implementation.

This study also provides valuable insights into applying LLMs to non-English medical documents. Despite being trained predominantly on English data, the LLM (ChatGPT-4o) exhibited trends in Korean—improved readability coupled with potential compromises in risk communication—that mirror findings from English-based studies [14]. This suggests some language-agnostic behaviors but also reinforces concerns that current models may prioritize fluency over domain-specific accuracy and medicolegal completeness across language [15]. These results highlight the need for developing and fine-tuning LLMs on high-quality, multilingual medical corpora, incorporating country-specific regulatory requirements to ensure both accessibility and compliance [9,16]. Future comparative studies across various languages and countries are needed.

Our findings should be interpreted with several limitations. Primarily, this study does not directly assess patient comprehension; therefore, the observed readability gains do not necessarily confirm improved patient understanding or better informed decision-making. Second, the use of only one LLM (ChatGPT-4o) without comparison to other models restricts conclusions about the optimal simplification approach. Furthermore, the focus on Korean language and liver resection consent forms limits generalizability to other languages, cultural contexts, and surgical procedures, which may have different informational needs and legal requirements. Finally, our evaluation relies on specific readability metrics and expert ratings, which may not fully capture all dimensions of effective communication or perfectly align with patient needs or legal sufficiency. Addressing these limitations through patient-centered assessments, comparative studies across LLMs and languages, and broader procedural scope represents crucial directions for future research.

### Conclusion

In summary, although LLMs hold promise for improving the accessibility of surgical consent forms, achieving this requires a careful balancing act. The gains in readability must be weighed against potential reduction in perceived content integrity, professional tone, and medicolegal robustness, particularly concerning risk information. Further research is essential primarily focusing on patient comprehension studies and encompassing validation against specific legal and ethical standards, comparisons across different LLMs and languages, and exploration of hybrid human-AI approaches to optimize both readability and fidelity in clinical settings.

## Abbreviations

AI: artificial intelligence
LLM: large language model
